# Health Tract, No. 87

**Published:** 1862-07

**Authors:** 


### WOMAN'S TRUE BEAUTY.

"I WAS glad to have it in my power to do any thing my husband wanted me to do," was the beautiful reply of a wife, long married, of wealth and position, when I asked her why, by over-taxing herself, she had induced great bodily suffering.

A man was terribly injured; a muslin bandage was essential to his safety; it was not at hand, and there was no time to run for it. A young woman present disappeared, and returned the next minute with the requisite article taken from her under-garment, and the poor soldier's life was saved.

On a bleak winter's night, a mere scrawl was handed in at the door, with a name known to fame; death was imminent. The patient was in a kind of out-house. back from the street. A solitary woman attended the unfortunate sufferer, silent, busy, anticipating every want, translating every gesture, almost before it was made. In the early morning, at noon, and in the dreary hours of darkness, she was always there; prompt, noiseless, vigilant, self-possessed. Day after day it was the same thing; and with all this, there was such a benignity in the whole demeanor, that I wanted to know her name and her relation to the patient, who had been abandoned by the dearest ties of humanity, and whose mental state was evidently as great a torture as that of the body. The tumultuous heavings of the mind and conscience were in sad unison with the ceaseless tossings of the emaciated frame, and the vain efforts of the restless, tearless eye to close itself in sleep. "I shall die if I don't sleep," was the constant, piteous exclamation! Lover and friend and daughter even kept sternly aloof from that miserable bed-side. She had heard of the hapless and abandoned sufferer, and for humanity's sake, supplied every want from her own purse, and continued so to do, for weeks and weary months, until death brought relief from the fearful combination of human sufferings. To do so much and for so long a time; to administer tireless personal attentions, and unstinted pecuniary aid to one so abandoned, without hope or possibility of reward, was the work of that angel of goodness, who has written so much and so sweetly in prose and verse—ALICE CAREY.

"My dear wife, I am hopelessly bankrupt," said a merchant when he entered his fine mansion, at the close of a day, all fruitless in his endeavor to save himself when men were crashing around him in every direction. "Tell me the particulars, dearest," said his wife calmly. On hearing them and his wants to save him, "Is that all?" and absenting herself a moment, returned with a book, from between the leaves of which she took out bank-note after bank-note, until enough was counted to fully meet all her husband's requirements. "This," said she, in reply to his mingled look of admiration and astonishment, "is what I have saved, for such a possible day as this, from your princely allowance for dressing myself, since we were married."

If every mother made it her ambition to mold her daughters' hearts in forms like these, who shall deny that many a suicide would be prevented; that many a noble-hearted man would be saved from a life of abandonment or a drunkard's dreadful death, and many families prevented being thrown upon society in destitution and helplessness, to furnish inmates for the jail, the poor-house, the asylum, and the hospital?

